# Twin Pregnancy—One Fœtus in Utero and the Other Extra Uterine*Read before the Nebraska State Medical Society, at Omaha, May 12th 1892.

**Published:** 1892-07

**Authors:** E. W. Martin

**Affiliations:** Fremont, Nebraska


### TWIN PREGNANCY.—ONE FŒTUS IN UTERO AND THE OTHER EXTRA UTERINE.*

BY DR. E. W. MARTIN, FREMONT, NEBRASKA.

Mrs. P., aged 40 years, of Scotch decent, having borne three children and had one premature labor. The eldest child aged 16, the youngest 5 years. A fœtus was prematurely de-delivered three years before the birth of the youngest child. She became pregnant again about the middle of February, 1890. The history of this case proves to be very peculiar.

Nothing remarkable or unusual having been observed in either of her former pregnancies.

On April 30th, I was called to see her, she having discharged her regular physician, by whom she had been attended for several weeks. When I first saw this woman, on superficial examination I thought it a case of septic peritonitis. The vital phenomena were suffering from general prostration and an unlimited degree of pain was manifest. I found her lying upon her back unable to move or be touched; emaciated and suffering great pain in the region of the ovaries, and particularly in the right hip. Absolute constipation had existed for 12 days, accompanied by excessive vomiting. The tongue was coated with a heavy fur in the center and normal color on its edge. She was having considerable metrorrhagia, which had begun a fortnight before. By vaginal and rectal examination, I diagnosed a retroverted, impacted, gravid uterus. By the knee and chest position, after two days effort, I was able in a great measure to replace the organ. Symptoms began to improve, the vomiting ceased, and by a copious injection per rectum, the bowels moved, and with a little encouragement they soon resumed some degree of regularity.

Her appetite returned, and within ten days the patient was up and walking about the house and yard. Immediately upon her effort to be on her feet, her right leg began to swell, and assumed alarming proportions. The foot, ankle and leg to the knee presented marked œdema. With but slight improvement, this state of thing continued until July

*Read before the Nebraska State Medical Society, at Omaha, May 12th 1892.

18th, when I was suddenly called, and found her with all symptoms of labor. It should have been stated that at intervals of every five or six hours, slight hemorrhages from the uterus had continued from the time I first saw her until the present. The labor symptoms now continued until the 23d, when a five months fœtus was discharged. The placenta presented the appearance of having been partially detached, which doubtless accounts for the continued hemorrhage. The placenta came away with the same pain that expelled the fœtus, when all pains ceased, followed by little hemorrhage.

For two days I irrigated the uterus with antiseptic lotions. My attention was called by the patient to the fact that she was having quickening, as though another child existed in the uterus. On placing my hand upon the abdomen, I was convinced beyond a doubt that her conjectures were not entirely groundless, as the movements of the child could be distinctly felt at this juncture. However, the patient got up within five days, the swelling disappeared from the limb, she gained in flesh and strength until the 17th of August, when pain began in the region of the right ovary, considerable pain in right hip, back and right limb; gave morphia to relieve pain. By digital examination, or by any other process, I was unable to say whether I had remaining ectopic pregnancy or not. The os was lax and flabby, and although I could pass my finger well up through, I could find nothing within reach, and doubting the propriety of exploring with the sound, I immediately wrote a history of the case and sent it to Dr. Thad A. Reamy, of Cincinnati, Ohio, asking him what he thought I had, whether double pregnancy, with more than one placenta, a bifid uterus or extra-uterine pregnancy. If the latter, he thought at this stage it must be of the so-called abdominal variety. He, like Lawson Tait, believed that primary abdominal pregnancy rarely if ever occurred; such cases usually being due to early rupture of the Fallopian tube, and escape of fœtus into the abdominal cavity. He advised me not to explore the uterine cavity, but to watch the case, as he believed it to be one simply of plural uterine pregnancy. Upon this theory I relied for a short time only, when I made my diagnosis—ectopic pregnancy. In the meantime, the ordinary symptoms had been going on, unmistakable quickening, placenta soufle, fœtal heart sounds and progressive increase in size. When I made my diagnosis, then trouble came.

Consultation was demanded and the ordinary routine indulged in. Dr. L. B. Smith of my city was called, and three M. D.'s from Lincoln, Nebraska, Drs. Peobles, Mitchell and Griffin. Suffice it to say, the case was so difficult of diagnosis that we were divided, some holding to the theory of a bifid uterus, and others to the ectopic theory. Dr. Peobles was not called until Sept. 25th. On Sept. 26 (next day,) rupture of the sac occurred, followed by collapse. Sept. 27th, at 8:30 P. M., death occurred.

On Sunday, Sept. 28th, I called all who were in consultation, and a post mortem was held, which developed the following: Extra-uterine pregnancy of seven and one-half months gestation, remaining after the expulsion of more than two months previously from the womb a fœtus of an uterine pregnancy.

The development of the two fœti would indicate that conception had taken place at the same time, and from all the circumstances, this theory is the most plausible.

Abdominal action displayed a ruptured chorion, and escape of fœtus and liquor amnii into the abdominal cavity and viscera.

The fœtus was easily removed from among the intestines.

The chorion was found to have many adhesions to both the parietes and viscera. Evidences of an old peritonitis having existed in the region of the right ovary, was patent. The adhesion developed the impossibility of a successful operation of laparotomy being performed at any time after the expulsion of the first fœtus. At no time previous could a correct diagnosis have been made. With a child in utero, no man would have risked his reputation, to say there was another also in the abdominal cavity. Another singular incident now presented itself. The umbilical cord was bifurcated, and led to two placentas. The first of small development, weighing about three or four ounces, had attached itself to the folds of the peritoneum about the left broad ligament. The other weighing two or three pounds, found its attachments in Douglas' cul-de-sac. It would seem, at the time of conception, that at least one of the ova in its transit from the ovary to the uterus, was arrested in that portion of the Fallopian tube passing through the walls of the uterus; there it became fructified and the development began. This would be called interstitial pregnancy. The post-mortem developed this to be true. As the muscular fibers of the uterus became stretched and distended, it formed the outer covering of the ovum, and accounts for its going to that long period of seven and one-half months before rupture, as it is very susceptible of distention, at least it proved so in this case. A thin layer of uterine muscular fibers was found surrounding nearly the whole of the sack, notwithstanding the fact that it is asserted by some of the most eminent authorities that no placenta having primarily attached itself to the tube or peritoneum, can attach itself to any other structure. How can we account for the firm attachment of the one in Douglas' cul-de-sac. Was that it's primary location? If not, how did it get there? The divisions and sub-divisions of ectopic pregnancy so found in most standard text-books, tend to confuse the medical mind, for such classifications are not sustained by facts correctly observed. It is not my purpose in this paper to speculate or philosophise, but have attempted to give you all the facts, as far as I was able, connected with this most interesting case. So far as I have looked up medical literature, there has been no case reported like it. In conclusion, it is but right to say that our subject closed the life of one of the most elegant and cultivated ladies of the West.

---

### ENGORGEMENT OF THE UTERUS-GONORRHÆ.

I have tried Sanmetto in engorgement of the uterus and found it better than anything I ever used. Also in a case of long standing gonorrhæ which gave permanent relief.

W. C. WOOD, M. D., Woodward, Ala.

